# Predominance of *Trichophyton soudanense* as Agent of Dermatophytoses in Cape Verdean School-Age Children

**DOI:** 10.3390/jof10100693

**Published:** 2024-10-04

**Authors:** Edmilson Emanuel Monteiro Correia, Marta Mota, Luciano Vagner Ascenção de Melo Veiga, Chantal Fernandes, Teresa Gonçalves

**Affiliations:** 1 CNC-UC—Center for Neuroscience and Cell Biology, University of Coimbra, 3004-504 Coimbra, Portugal; edcorreia17@gmail.com (E.E.M.C.); marta.mota83@gmail.com (M.M.); 2 CIBB—Centre for Innovative Biomedicine and Biotechnology, University of Coimbra, 3004-504 Coimbra, Portugal; 3 FMUC—Faculty of Medicine, University of Coimbra, 3004-504 Coimbra, Portugal; 4 Health Delegation of São Salvador do Mundo, Achada Igreja 7329, Cape Verde; luciano.veiga@ms.gov.cv

**Keywords:** dermatophytoses, Cape Verde, school-age children, dermatophytes, *Trichophyton soudanense*

## Abstract

Dermatophytoses are infectious skin diseases of public health importance because of their transmissibility and high prevalence, especially among children. This is the first study aiming to estimate and report the burden of dermatophytoses on school-age children on the island of Santiago in Cape Verde, an African country that is an archipelago. A total of 249 students attending the afternoon shift of three elementary schools in the city of Achada Igreja were examined. Of these, 60 had suspected lesions of dermatophyte infection. However, from the samples collected from these 60 students, including hair, nails, and skin scrapings, only 18 dermatophyte isolates were obtained, corresponding to a point prevalence of 7.2%. Morphological species identification demonstrated three different species: *Trichophyton soudanense*, *Trichophyton rubrum*, and *Trichophyton violaceum*; re-identification by sequencing the internal transcribed spacer (ITS) 1 and 2 regions of ribosomal DNA, and the 5.8S rDNA encoding gene (ITS-5.8S region), revealed *T. soudanense* as the most prevalent species, with only one case of *T. rubrum*. This is the first epidemiological data describing dermatophytoses and dermatophytes in Cape Verde among school-age children on one of the archipelago islands. It reinforces the need of using culture and accurate identification methodologies when gathering epidemiological data on dermatophytoses.

## 1. Introduction

Dermatophytoses are common contagious skin, nail, and hair diseases caused by fungi, known as dermatophytes [1]. They represent an important global public health problem despite being more prevalent in hot and humid regions [2,3]. In Africa, it has always been frequent to diagnose dermatophytoses, mainly in school-age children living in poor areas [4]. These are multifactorial skin infectious diseases, including geographic, climatic, and socio-economic factors [5], also with evidence of genetic predisposition [6,7]. Due to their non-lethality, they have earned poor attention compared to invasive fungal infections, despite their emotional and psychological consequences on patients together with their economic impact [8,9]. 

The dermatophytes are ubiquitous fungi belonging to the family Arthrodermataceae, which are distributed among nine distinct genera: *Trichophyton*, *Microsporum*, *Epidermophyton*, *Arthroderma*, *Lopophyton*, *Nannizia*, *Ctenomyces*, *Guarromyces*, and *Paraphyton* [10]. Although not all species are pathogenic, all are keratin hydrolyzers. Ecologically, dermatophytes are classified as geophilic (soil saprophytes), some of which can be transmitted to humans or animals, most often through trauma or soil contamination, as zoophilic pathogens adapted to one or more species of animals, causing infections in humans accidentally and, as anthropophilic, restricted to humans, yet isolated in some cases of infections in animals. Initially, soil saprophytes (geophilic) adapted to animals (zoophilic) and humans (anthropophilic), making them their main reservoirs [2,11,12,13]. The group of anthropophilic species stand out as the most important in public health, due to their high interpersonal transmission and ability to inhibit or evade the immune system, especially in chronic infections, and the occurrence of cases of in vitro resistance or failure of the antifungal therapies [14,15]. While some of the agents of dermatophytoses are globally distributed, others are restricted to certain regions or described as being eradicated in other zones of the globe [16]. Their prevalence can change over time, and one of the main factors contributing to this change is the displacement of populations (migration, mass tourism, and refugees’ reception) [2,17,18,19]. 

In European and North American countries, several recent studies reported changes in the epidemiological profile of the agents of dermatophytoses [20]. For example, after presumedly being eradicated in Germany, *Microsporum audouinii*, an endemic species from Africa, was found in this European country in sporadic cases of tinea capitis, and the same was observed in other European and American countries. These outbreaks, especially among schoolchildren and students, were associated with African immigrants or refugees. In the past three decades, *Trichophyton rubrum* has become the most common dermatophyte in Northern and Central Europe, while *Microsporum canis* ranks as the more prevalent in the south and Mediterranean states [16,21]. In American countries, including the United States of America, Mexico, and Puerto Rico, despite a decrease in *T. rubrum* prevalence and an increase in *Trichophyton tonsurans* prevalence, *T. rubrum* is still the most isolated species, followed by *Trichophyton interdigitale* [21]. In Africa, *Trichophyton violaceum*, *Trichophyton soudanense*, and *M. audouinii* are the predominant species, varying from region to region and country to country [4,22,23]. In the northern region of Africa, where dermatophytoses are less observed, infections have been widely reported with a higher frequency of tinea capitis, onychomycosis, tinea pedis, and tinea corporis despite the improvement in living conditions and hygiene. In Algeria, the predominant dermatophytoses are observed in the nail, feet, and inguinal folds; these are caused essentially by *T. rubrum*. Tinea capitis is caused mainly by *T. mentagrophytes* and *M. audouinii* in the south [4,22]. In Tunisia and Morocco, *T. violaceum* and *M. canis* are the most common agents of tinea capitis; in these same countries, *T. rubrum* is the main isolated species, especially in toenail infections [4,22]. In urban areas of Egypt, *T. rubrum* and *T. interdigitale* are prevalent, causing onychomycosis, followed by *Microsporum* spp. and *T. tonsurans,* as agents of tinea capitis. In rural areas, with increased probability of contact with animals, *Trichophyton verrucosum* and *T. violaceum* were the most isolated species [4,22]. In Sudan, a strong presence of *T. verrucosum*, *T. mentagrophytes*, and *Trichophyton schoenleinii* were reported [22]. According to Nweze and collaborators [23], in most of the countries in the eastern and southern regions of Africa, *T. violaceum*, *Trichophyton tonsurans*, and *T. verrucosum* are the most prevalent agents of dermatophytosis. In Western and Central Africa, *T. soudanense* was the predominant species isolated from the scalp, followed by *M. audouinii*, *T. mentagrophytes*, and *T. tonsurans* [24,25]. In Asia, the world’s largest and the most populous continent, *T. rubrum* and *T. mentagrophytes* were the most prevalent dermatophytes identified [21]. It is worthwhile to mention that in most of the reports cited, the identification of dermatophytes was obtained using classical methods. Most recent studies used molecular methods for the detection, accurate identification, and characterization of the species of dermatophytes most commonly involved in human and animal infections, recognizing the importance of accurate identification in the prevention and treatment of dermatophytoses but also contributing for the increased quality of the reported epidemiological data [16].

In Cape Verde, an African archipelago, there are no studies regarding epidemiological data on dermatophytoses, although clinicians’ informal reports describe a high number of suspected cases especially among school-age children. This lead us to conduct this study, with the objective of gathering data on the profile and prevalence of infections by dermatophytes at three primary schools in Cape Verde.

## 2. Materials and Methods

### 2.1. Selection of Patients and Ethical Issues 

This cross-sectional study was carried out in three rural schools, with low socio-economic status, in the city of Achada Igreja, a small town located in the central region of the island of Santiago, Cape Verde, 23 km northwest of the capital city of Praia. The population data were obtained at the Cabo Verde National Institute of Statistics. The municipality consists of one city and 21 villages, with a total number of 8608 residents, 528 of them studying in three elementary schools. This study was approved by the Ethics Committee of the participating institutions (Cape Verde National Data Protection Commission, authorization number 237/2019, and the Faculty of Medicine of the University of Coimbra, Portugal, Ethics Committee authorization number 014-CE-2020). Informed consent was obtained from the guardians together with the children’s assent to participate. 

### 2.2. Sample and Data Collection

The samples were collected during the first three weeks of March 2020. Of the total 528 students attending the school, only 249 students in the afternoon shift were enrolled in this study. The physical examination of the students included signs and symptoms such as itching, spots, scaling, hair loss, kerion, scabs, and pustules. A total of 60 children were selected based on the skin lesions. Their family history was also taken into consideration for the clinical diagnosis of dermatophytoses. Sociodemographic issues and hygiene habits were obtained in the form of a questionnaire (Supplementary Material, Table S1). Skin, hair, or nail samples were collected according to standard procedures from students with clinically suspected dermatophytosis. Briefly, samples were collected by scrapping after antisepsis of the affected anatomical site, using 70% ethanol, to minimize contaminants. The samples were transferred and kept in sterilized containers until laboratory processing. Before collection, the lesions were documented with photos.

### 2.3. Culture and Identification of Dermatophytes

The culture and identification of dermatophytes were performed as described before [26]. The clinical samples were cultivated on Sabouraud dextrose agar (SDA) added with chloramphenicol (50 μg/mL) and cycloheximide (300 μg/mL). After culture, with up to thirty days of incubation at 30 °C, the isolated dermatophytes were first identified based on their macroscopic characteristics, in SDA and in PDA (potato dextrose agar). The identification was further confirmed using molecular tools by sequencing the internal transcribed spacer (ITS) regions 1 and 2 of ribosomal DNA (rDNA). For that, DNA was extracted from 7-day-old cultures, using the Instagene Matrix extraction kit (Bio-Rad Laboratories, Hercules, CA, USA). Briefly, in a 1.5 Eppendorf tube, 200 μL of the InstaGene Matrix suspension, containing beads, was introduced, to which a fragment of the mycelium was added. Tubes were incubated at 56 °C for 30 min in a thermoblock, vortexed after incubation, and re-incubated at 100 °C for 8 min before centrifugation at 12,000 rpm for 3 min. The supernatant was carefully pipetted and DNA concentration and purity were analyzed using Nanodrop^®^. Universal primers ITS1 (3′-TCCGTAGGTGAACCTGCGG) and ITS4 (TCCTCCGCTTATTGATATGC) were used to amplify the ITS-5.8S regions of the rDNA gene cluster. The amplified DNA was purified using the commercial NucleoSpin^®^ Extract II kit and sent elsewhere for sequencing (LGC Genomics, Berlin, Germany). The identification was obtained by comparing the overlapping sequences of the forward and reverse reads in the NCBI database using a BLAST Search. The nucleotide sequences were then submitted to the NIH genetic sequence database (GenBank^®^, NCBI) (details in Supplementary Materials, Tables S2–S4).

## 3. Results

In the three schools selected for this study, a total of 249 students attended the first until the sixth school years, in the afternoon shift. Among these 249 students, 134 (53.8%) had skin diseases; from these, only 60 students were suspected or had a clinical diagnosis of dermatophytosis. These 60 students corresponded to 44.8% of the children with suspected skin diseases of the three schools, and to 24.1% of the total school population in the afternoon shift. The clinical examination of the students showed mainly non-inflammatory conditions, scales, and spots similar to seborrheic dermatitis, the desquamation of the gray spots, kerion, crust, pustules, hair breakage and loss, nail destruction, and itching in most cases. Of these, 24 (40.0%) were girls, and 36 (60.0%) were boys, aged between 7 and 16 years (Table 1; Supplementary Materials, Table S1). All students were African-born in Cape Verde, living in villages next to the schools, without closer contact with foreign people. The students from the three schools had similar socio-economic settings; most of them had fair hygiene practices, with three to seven showers per week and with daily contact with animals, husbandries, and farming activities; however, they had no hygiene procedures immediately after these activities. Of the total, 46.7% (28) were usually barefoot and 60.0% (36) shared clothes, shoes, a bath towel, a bed, and sheets with other members of the family (Table 1).

After culture of the collected samples, only 18 students had a confirmation of the clinical diagnosis, since only for these was it possible to isolate and to identify dermatophytes. Among the students with confirmed dermatophytosis, 10 (55.6% of the total 18 with a confirmed diagnosis) were not receiving any treatment, and 7 (38.9%) received topical therapy, essentially with ketoconazole. One child (5.6%), aged 7, diagnosed with tinea capitis, was receiving griseofulvin (Table 2).

**Figure 1:**
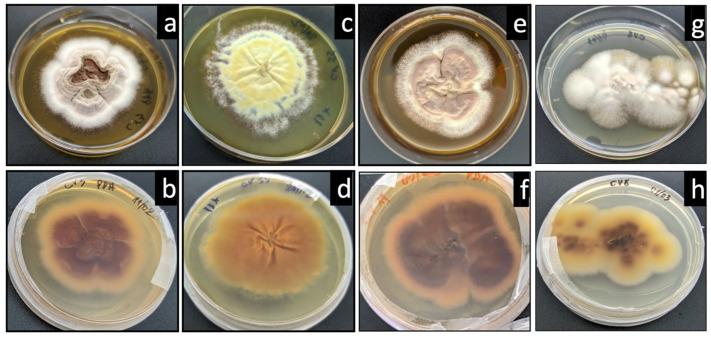
Macroscopic presentation of isolates suggestive of *T. soudanense*, confluent, velvety, yellow, and beige colonies (in potato dextrose agar—PDA), surface (**a**,**c**,**e**,**g**) and reverse (**b**,**d**,**f**,**h**).

**Figure 2:**
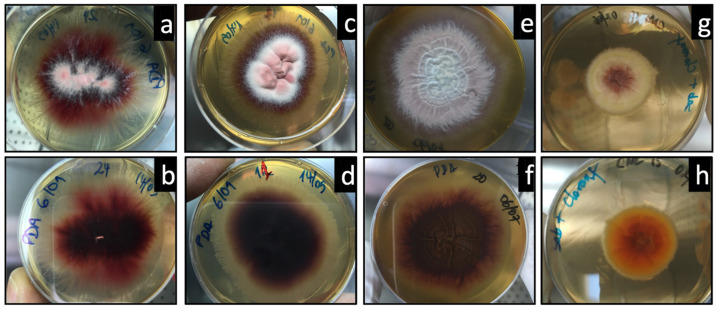
The macroscopic presentation of isolates suggestive of *T. rubrum* (**a**–**d**), white to reddish colonies, mostly flat to slightly raised, suede-like, and appearing as red on the reverse, and suggestive of *T. violaceum* (**e**–**h**), glabrous or serous colonies, folded and violet with white sectors and deep violet on the reverse (in potato dextrose agar—PDA). Colony surface—top row; colony reverse—bottom row.

After culture, and since this study had a focus on dermatophytes, the isolates macroscopically resembling dermatophytes were identified using two methodologies. First, using macroscopic colony morphology, in three species, the isolates were presumptively identified in three species. The glabrous colonies observed after one week (rapid growth), presenting ridged surfaces, beige or yellowish, and with a velvety texture (Figure 1), were suggestive of *T. soudanense*. Colonies with a suede surface and with reverse pigmentation, yellow-brown or wine red, were suggestive of *T. rubrum* (Figure 2). 

The colonies with a flat spiral relief, with a violet edge, or purple colonies with a white outline, and a suede-like surface texture (Figure 2), were suggestive of *Trichophyton violaceum*. 

The second strategy for the identification of the isolates was performed using a molecular approach, by the amplification and sequencing of the ITS-5.8S region of the rDNA. The results obtained showed that there was no total match (Table 2; detailed sequences and results in Supplementary Materials, Table S4). In fact, based on the sequences of ITS-5.8S rDNA, 18 isolates were identified as *T. soudanense* and 1 as *T. rubrum*, an infection of the nail (Table 2). A group of five isolates, two isolated from skin lesions (CV53, CV59), one from the nail (CV56), and two from the face (CV57, CV61), although with colony morphologies resembling dermatophytes, could not be identified using the classical morphological identification. The sequence of the ITS-5.8S region of the rDNA revealed that these were non-dermatophyte filamentous fungi, *Chaetomium convolutum* (three isolates), *Arthrinium marii* (one isolate), and *Aureobasidium pullulans* (one isolate). All the isolates confirmed as dermatophytes are under the process of deposition at the clinical fungal culture collection of the University of Coimbra (Clinical Yeast Culture Collection University of Coimbra, CYCCUC; https://www.uc.pt/en/fmuc/cyccuc/, accessed on 24 August 2024). 

**Figure 3:**
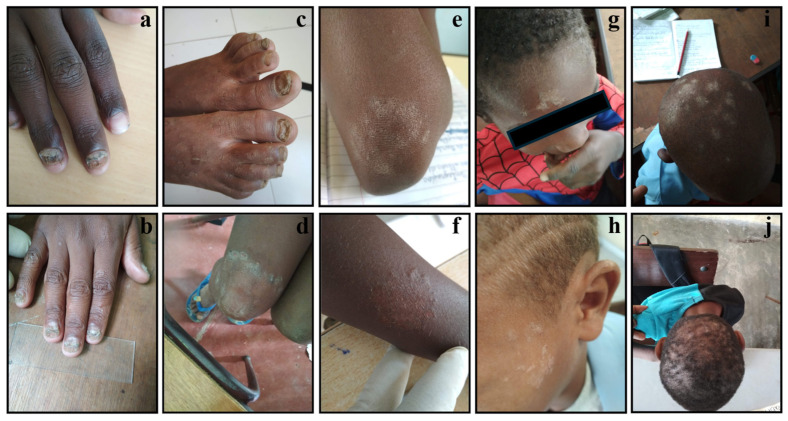
Clinical aspects of dermatophytoses by *T. soudanense* in elementary school children in Achada Igreja, Picos, Santiago Island, Cape Verde. Legend: (**a**–**c**) illustrate nail lesions, (**d**–**f**) glabrous skin lesions (tinea corporis), (**g**,**h**) tinea faciei, (**i**) scalp lesions, and (**j**) tinea capitis.

The skin lesions observed during this study were documented with photos. Figure 3 shows the lesions from which *T. soudanense* was isolated. These were observed in glabrous skin, corresponding to 72.2% (13/18), in the body, hand, leg, arm, and face; in the nails (4/18; 22,2%); and in the scalp (1/18; 5.6%). This latter case constituted the only child diagnosed with tinea capitis. Of these children infected with dermatophytes, 12/18 (66.7%) were boys aged between 7 and 14 years and 6/18 (33.3%) were girls aged between 8 and 11 years. 

In Figure 3, the lesions due to *T. soudanense* can be observed, either in the skin, nails, or scalp. Only one student had a positive culture for *T. rubrum*, in a nail lesion. Although all the selected students have daily contact with animals, pets, and cattle, no zoophilic species were isolated. 

Regarding the prevalence, it can be considered that in the total population of students of the three schools (249 students attending the afternoon shift), the point prevalence of dermatophyte infection was 18/249 (7.2%).

## 4. Discussion

The incidence of dermatophytoses is becoming increasingly common in developed economies [17,18,27], especially in Africa [4,23,24]. In Cape Verde, despite the clinical empirical awareness of a tendency for a high incidence of dermatophytoses, especially among school-age children in rural areas, there are no reports on dermatophytoses and on the agents. To our knowledge, this is the first report on dermatophytoses ever carried out in Cape Verde. This study was conducted in school-aged children attending three elementary schools in the city of Achada Igreja, on the island of Santiago. Among the 249 students in the afternoon shift, 60 children were clinically considered has having lesions compatible with dermatophytosis, but mycological investigations allowed us to confirm dermatophyte infections for only 18 of them, with a point prevalence of 7.2%, a value lower than that described by several other studies in pediatric populations in Africa, with prevalence values of 20% of school-age children in West Africa, and prevalence values that can range from 10% to more than 70% in other regions of Africa [4]. The clinical examination of the students showed mainly non-inflammatory conditions, such as scales, and spots similar to seborrheic dermatitis, the desquamation of the gray spots, kerion, crusts, pustules, hair breakage and loss, nail destruction, and itching. However, similar lesions can be caused by other organisms, making the clinical diagnosis merely presumptive [28,29,30,31]. Most of the selected children with a suspicion of dermatophyte infection, although with the habit of showering, had inadequate hygiene after barefoot activities, such as walking long distances to school and agricultural or animal husbandry activities. Children with such a lifestyle were previously described as having higher susceptibility of getting dermatophytoses [2,32]. We also report insufficient health-seeking practices, since even if they visited a family practitioner and an antifungal was prescribed, there was no monitoring and follow-up of the outcome of the infection/lesion; some were under topical treatment with ethnomedical products (results not shown). 

Two different identification methodologies were used: the morphological classical identification of dermatophytes and molecular biology, by ITS-5.8S rDNA sequencing. Based on the observation of macroscopic and microscopic morphology, three species were identified, *T. soudanense*, *T. violaceum*, and *T. rubrum*. Re-identification using molecular biology showed only one *T. rubrum* isolate, with the remaining isolates confirmed to be *T. soudanense* after sequencing of the ITS–5.8 S region of the rDNA (and four isolates excluded as dermatophytes). The taxonomy and the correct identification of the species included in the *T. rubrum* complex have been the subject of several studies. Some authors considered *T. soudanense* and *T. violaceum* as synonyms [33], while others, using MALDI-TOF MS, recognized these as different species of the *T. rubrum* complex [34,35]. Nevertheless, the utilization of ITS phylogeny proved that *T. soudanense* is an independent species [35,36]. A recent phylogenomic approach revealed limitations in ITS sequencing to distinguish between the three main species of the *T. rubrum* complex, *T. soudanense*, *T. violaceum*, and *T. rubrum*, and describe other genes more suitable to distinguish between species, a ubiquitin-protein transferase, and an MYB DNA-binding domain-containing protein [37]. 

*T. soudanense* is an anthropophilic dermatophyte endemic in Africa [4], primarily responsible for tinea capitis and tinea corporis in children, and more rarely in adults. It is also sporadically isolated in countries with cultural, social, or economic relations with endemic areas, across Europe and North America [18,27]. The prevalence of *T. soudanense* in this study corroborates data from several other studies reporting this species as a highly prevalent species of *Trichophyton* in the central and western regions of Africa, with most of the studies focused on cases of tinea capitis [24,25,38,39,40,41], whereas we report *T. soudanense* as an infecting agent in the skin, nails, and scalp. However, differently from the findings obtained in West Africa, we did not find *M. audouinii* [40]. The fact that *T. soudanense* has a high epidemiological rate of inter-human transmission, and that the children included in this study had direct contact with each other, is suggestive of the contagious nature of these infections. However, since the children had daily contact with soil and animals, one could easily expect to find a predominance of geophilic or zoophilic species. This trend of the switch between zoophilic and anthropophilic dermatophytes was reported by others in India [32]. In fact, people with dermatophytoses can easily spread the infection to others by direct or indirect contact through shared surfaces and equipment and the persistence within the same physical environment may represent opportunities for reinfection and disease spread [42,43]. Moreover, since this study was held on one of the islands of the archipelago, the predominance of a species might reflect restricted contact, lowering the biodiversity of the agents of dermatophytoses. This was also observed in another epidemiological study of dermatophytoses on an African island, in Ethiopia [44], where *T. violaceum* was the predominant species among the mycological specimens. This is not the case on a bigger island, Crete in Greece, that receives thousands of tourists and immigrants, and where *M. canis*, *T. rubrum*, and *T. mentagrophytes* were the three most represented species [45].

The results obtained in this work reinforce that the agents responsible for dermatophytoses are difficult to identify based on clinical manifestations and on morphological characteristics, with molecular biology techniques assuming an important role in increasing the accuracy, as reported before [26,31]. The accurate identification of dermatophytes is essential for the efficient management of this public health problem [4,46], helping local health providers to differentiate between dermatophytic and non-dermatophytic superficial infections, to identify potential sources of infection, to draw preventing transmission strategies, and to implement the appropriate antifungal therapies [20]. 

As all the participants in this study were nationals, with no contact with immigrants, it is legitimate to conclude that the dermatophyte species found in this study are endemic. It was left to elucidate whether, in each school, the infections were caused by a single strain that persisted in the population and was transmitted between students or by different strains. Molecular typing of strains could indicate these important epidemiological issues [47,48,49]. Moreover, specific aspects such as morphological issues associated with the *T. soudanense* synonym, *Trichophyton gourvilii*, were not reported, although the isolates are available for future studies. Although our study is limited in size, it is a clinical and public health asset in the archipelago of Cape Verde, providing complementary information to the available epidemiological data on the prevalence of dermatophyte infection in Africa. Nevertheless, it should be mentioned that our study, like many others, is restricted to the isolation of dermatophytes causing infection, not taking into account the passive colonization by these fungi [50], which by itself constitutes an important epidemiological issue [51]. Moreover, it can be argued that the low prevalence might be due to the use of antifungal treatments, using conventional antifungals or ethnomedical products, leading to total fungal recovery impairment.

## 5. Conclusions

As a first approach to the epidemiology of infections by dermatophytes in the school-age pediatric population of Cape Verde, this study shows that the point prevalence of these infections is lower than that observed in other regions of Africa, although some limitations can be pointed such as complementing the information gathered with a screening for asymptomatic colonization by dermatophytes, and/or further using molecular biology to detect dermatophytes directly in the lesion specimens, independently of dermatophyte culture. It also shows the predominance of *T. soudanense*, an anthropophilic dermatophyte, allowing us to draw the conclusion that this infection is transmitted between the children at school and/or in the household context, although further studies are needed to prove this hypothesis. It can also be concluded that to gather accurate epidemiological data on dermatophytoses, it is mandatory to complement the clinical diagnosis, regarding the clinical presentations of the lesions, with laboratory studies using both classical procedures, including culture and morphological and microscopic identify, but also providing molecular-based identification methodologies. These will help in the exclusion of suspected lesion-like dermatophytosis and in the accurate characterization of the agents responsible for the infections.

## Figures and Tables

**Table 1:** Sociodemographic data of the studied population.

		Number/Percentage ^1^		
			Dermatophytosis ^1^	
			Yes	No
Mean age		10.4		
Sex	Female	26 (40.0%)	6 (10.0%)	18 (30%)
	Male	36 (60.0%)	12 (20.0%)	24 (40.0%)
Lesion localization	Scalp	5 (8.3%)	1 (1.7%)	4 (6.7%)
	Ear	1 (1.7%)	0	1 (1.7%)
	Hand	4 (6.7%)	2 (3.3%)	2 (3.3%)
	Toenail	1 (1.7%)	0	1 (1.7%)
	Leg	6 (10.0%)	2 (3.3%)	4 (6.7%)
	Arm	6 (10.0%)	2 (3.3%)	4 (6.7%)
	Body	10 (16.7%)	6 (10.0%)	4 (6.7%)
	Foot	1 (1.7%)	0	1 (1.7%)
	Interdigital	4 (6.7%)	0	4 (6.7%)
	Nail	15 (25.0%)	4 (6.7%)	11 (18.3%)
	Face	5 (8.3%)	1 (1.7%)	4 (6.7%)
	Back	1 (1.7%)	0	1 (1.7%)
	Skin	1 (1.7%)	0	1 (1.7%)
Barefoot		28 (46.7%)	9 (15.0%)	19 (31.7%)
Shared items ^2^		36 (60.0%)	11 (18.3%)	25 (41.7%)
Shower/week	3	12 (20.0%)	3 (5.0%)	9 (15.0%)
	4	20 (33.3%)	7 (11.7%)	13 (21.7%)
	5	16 (26.7%)	4 (6.7%)	12 (20.0%)
	6	4 (6.7%)	1 (1.7%)	3 (5.0%)
	7	8 (13.3%)	3 (5.0%)	5 (8.3%)

^1^ Percentages of the 60 selected students in total with a lesion suspected of infection by dermatophytes; ^2^ this includes sharing with other members of the family the following items: clothes, shoes, bath towels, a bed, and sheets.

**Table 2:** Isolation and identification of dermatophytes from lesions clinically suspected as dermatophytoses and corresponding NCBI accession number.

			Fungal Identification		
Sample	Body Site	Antifungal Treatment	Morphology	ITS-5.8S Sequencing	NCBI Accession Number
CV3	Body	Ketoconazole	*T. violaceum*	*T. soudanense*	OR168629
CV4	Hand	Ketoconazole	*T. soudanense*	*T. soudanense*	OR185564
CV8	Nail	None	*T. soudanense*	*T. soudanense*	OR206379
CV10	Leg	None	*T. soudanense*	*T. soudanense*	OR185566
CV11	Body	Ketoconazole	*T. rubrum*	*T. soudanense*	OR185565
CV12	Arm	None	*T. violaceum*	*T. soudanense*	OR185567
CV15	Face	Ketoconazole	*T. violaceum*	*T. soudanense*	OR206381
CV20	Body	None	*T. violaceum*	*T. soudanense*	OR185597
CV24	Leg	None	*T. rubrum*	*T. soudanense*	OR185598
CV30	Body	None	*T. violaceum*	*T. soudanense*	OR185599
CV42	Arm	Ketoconazole	*T. soudanense*	*T. soudanense*	OR185600
CV45	Body	Ketoconazole	*T. soudanense*	*T. soudanense*	OR185601
CV47	Body	None	*T. soudanense*	*T. soudanense*	OR185602
CV50	Toenail	None	*T. rubrum*	*T. soudanense*	OR185603
CV52	Hand	Clotrimazole	*T. rubrum*	*T. soudanense*	OR185607
CV55	Nail	None	*T. rubrum*	*T. rubrum*	OR249962
CV58	Nail	None	*T. soudanense*	*T. soudanense*	OR186345
CV60	Scalp	Griseofulvin	*T. soudanense*	*T. soudanense*	OR206380

## Data Availability

The nucleotide sequences of the ITS–5.8 S region of the rDNA of the clinical isolates are available at the NIH genetic sequence database GenBank^®^, NCBI.

## Supplementary Materials

The following supporting information can be downloaded at: https://www.mdpi.com/article/10.3390/jof10100693/s1, Table S1: Characterization of the population of students included in this study; Table S2: Mixtures for the ITS-5.8 S PCR reaction.; Table S3: Number and time of each cycle of the PCR reaction; Table S4: Molecular identification of the dermatophytes isolates and corresponding accession number.
