# Supplementary material for: Predominance of Trichophyton soudanense as Agent of Dermatophytoses in Cape Verdean School-Age Children
Source: J Fungi (Basel). 2024 Oct 4;10(10):693. doi: 10.3390/jof10100693 (PMC11508676; doi:10.3390/jof10100693)
Supplement: Supplementary file 1 [file jof-10-00693-s001.zip › jof-3123064-supplementary.pdf]

Supplementary material

Title: Predominance of *Trichophyton soudanense* as agent of dermatophytoses in Cape Verdean school-age children

Journal: Journal of Fungi

Authors:

Edmilson Emanuel Monteiro Correia (Orcid 0000-0003-3265-954X) [1], Marta Mota (Orcid 0000-0003-4711-4817)[1,2,3], Luciano Vagner Ascenção de Melo Veiga[4], Chantal Fernandes (Orcid 0000-0001-7865-9946)[1,2,*], Teresa Gonçalves (Orcid 0000-0001-9347-0535) [1,2,3,*].

Afilliations:

[1] CNC -UC – Center for Neuroscience and Cell Biology, University of Coimbra, Portugal

[2] CIBB—Centre for Innovative Biomedicine and Biotechnology, University of Coimbra, Portugal

[3] FMUC - Faculty of Medicine, University of Coimbra, Portugal

[4] Health Delegation of São Salvador do Mundo, Cabo Verde

***Correspondence:** Teresa Gonçalves; Email: tmfog@ci.uc.pt; xantal@gmail.com

1. Characterization of the population of students

Table S1. Characterization of the population of students included in this study

Sample ID	Age/Sex	School	Scholar Level	Localization	Animal Contact	Shower /Week	Barefoot	Sharing Clothes
CV1	9/m	Achada igreja	4	Scalp	No	4	No	Yes
CV2	9/m		4	Ear	No	5	No	No
CV3	11/f		5	Body	Yes	4	Yes	Yes
CV4	11/f		6	Hand	Yes	4	Yes	Yes
CV5	10/f		5	Toenail	Yes	3	Yes	No
CV6	10/f		5	Nail	Yes	3	Yes	Yes
CV7	12/m		6	Back	No	5	No	No
CV8	10/f		4	Nail	Yes	7	Yes	Yes
CV9	12/m		6	Leg	Yes	3	Yes	Yes
CV10	12/m		6	Leg	Yes	3	Yes	Yes
CV11	12/m		6	Body	Yes	4	No	No
CV12	12/m		5	Arm	Yes	3	No	No
CV13	13/f		6	Nail	Yes	5	No	No
CV14	10/m		4	Body	Yes	3	No	No
CV15	11/m		5	face	Yes	5	Yes	Yes
CV16	10/f		4	Nail	Yes	7	No	Yes
CV17	10/m		4	Arm	Yes	5	Yes	Yes
CV18	10/f		5	Foot	No	4	No	Yes
CV19	12/m		6	face	Yes	6	No	Yes
CV20	14/m		6	Body	Yes	4	No	No
CV21	7/m	Chão Rodrigues	2	face	Yes	5	Yes	Yes
CV22	11/m		4	Nail	Yes	5	Yes	Yes
CV23	10/f		4	Nail	No	4	No	Yes
CV24	11/m		5	Leg	Yes	6	No	Yes
CV25	12/m		6	Interdigital	Yes	4	No	No
CV26	14/m		5	Hand	Yes	4	No	No
CV27	11/f		5	Body	Yes	5	No	Yes
CV28	10/f		4	Interdigital	Yes	7	Yes	No
CV29	10/f		5	Nail	Yes	5	No	Yes
CV30	10/f		5	Body	Yes	3	Yes	Yes
CV31	12/m		6	Arm	Yes	4	Yes	Yes
CV32	10/f		4	Nail	Yes	4	No	No
CV33	10/m		4	Nail	Yes	3	No	Yes
CV34	11/m		5	Arm	Yes	3	Yes	Yes
CV35	9/m		4	Interdigital	No	5	Yes	Yes
CV36	9/f		4	Interdigital	No	4	Yes	Yes
CV37	13/m		6	Hand	Yes	5	No	Yes
CV38	10/m		5	Leg	Yes	3	Yes	Yes

CV39	10/m		5	Leg	Yes	5	Yes	Yes
CV40	10/m		5	Body	Yes	7	Yes	No
CV42	10/f		4	Arm	Yes	4	No	No
CV43	11/f		4	Body	No	5	No	Yes
CV44	15/m		6	Leg	Yes	4	No	No
CV45	12/m		6	Body	Yes	4	Yes	No
CV46	10/f		4	Scalp	No	3	No	No
CV47	10/m		4	Body	Yes	7	No	Yes
CV48	11/m		5	Scalp	Yes	4	No	Yes
CV49	13/m		5	Arm	Yes	3	No	No
CV50	16/m		6	Nail	Yes	7	No	No
CV51	8/f		3	Nail	No	6	Yes	Yes
CV52	8/f	Achada Leitão	3	Hand	Yes	4	Yes	Yes
CV53	8/f		3	Scalp	Yes	7	Yes	Yes
CV54	8/f		3	Nail	Yes	4	Yes	No
CV55	7/m		3	Nail	Yes	5	No	No
CV56	7/f		3	Nail	Yes	4	Yes	Yes
CV57	8/f		3	Face	Yes	4	Yes	No
CV58	7/m		3	Nail	No	5	No	Yes
CV59	7/m		3	Skin	No	6	No	No
CV60	7/m		3	Scalp	Yes	5	Yes	Yes
CV61	11/m		5	Face	Yes	7	No	No

2. Identification of dermatophytes species by sequencing the ITS-5.8S regions of the rDNA gene

Extraction of genomic DNA from filamentous fungi

DNA extraction was performed using the commercial extraction kit, Instagene Matrix (Bio-Rad Laboratories, Hercules, CA, USA), with slight modifications. Extraction was done from 7 day-cultures grown on Potato dextrose agar (PDA) medium. In a 1.5 Eppendorf tube, 200 µ1 of the InstaGene Matrix suspension, containing beads, was introduced, to which a fragment of the mycelium was added with the help of a fork and placed in the tube with a sterile pipette tip. Tubes

were incubated at 56 °C for 30 min in a thermoblock, vortexed after incubation, and re-incubated at 100°C for 8 minutes before centrifugation at 12,000 rpm for 3 min. Then, 100 µl of the supernatant was carefully pipetted to avoid carrying beads.

DNA preparation and PCR Reaction

The concentration of the extracted DNA was measured using Nanodrop®. The samples were diluted to obtain 20 ng/µL of DNA. Aliquots were stored at -20°C until further use. The PCR mixtures were prepared in 0.2 mL reaction tubes for a final volume of 25 µl, as shown in **Table S2**.

Table S2. Mixtures for the ITS-5.8 S PCR reaction

**Component**	**Volume** (µl)	**Final Concentration**
**DNA**^{se} Free Water	**Volume to make 20 µl**	**1x**
5X Phusion HF Buffer	**4 µl**	
10mM dNTPs	**0.4 µl**	**200 µM each**
Forward Primer (ITS1)	**0.5 µl**	**0.5 µM**
Reverse Primer (ITS4)	**0.5 µl**	**0.5 µM**
Template DNA	**5 µl**	**20 ng/µl**
DNA Polymerase	**0.2 µl**	**0.02 U/ µl**

In the present work, the universal primers ITS1 (TCCGTAGGTGAACCTGCGG) and ITS4 (TCCTCCGCTTATTGATATGC) were used. In addition to amplifying the ITS1 and ITS2 regions

from total DNA extracted from the fungal isolates, they also amplify the 5.8S subunit of the fungal rDNA gene cluster.

The amplification reaction was carried out in a thermocycler, under the conditions shown in Table S3. After amplification, the amplification products were observed after electrophoresis with a 1.5% agarose gel. The bands corresponding to each sample were cut, and transferred to 1.5 ml Eppendorf to be purified, using the NucleoSpin®Extract II kit. Purified DNA samples were stored at 4°C, until sent for sequencing elsewhere. PCR products were sent to LGC Genomics (Germany, Berlin) for sequencing.

Table S3. Number and time of each cycle of the PCR reaction

**Cycle Step**	**3 Step Protocol**		
	Temp.	Time	Cycles
**Initial Denaturation**	98°C	30s	1
**Denaturation**	98°C	10s	
**Annealing**	56°C	10s	35
**Extension**	72°C	The 30s	
**Final Extension**	72°C	5min	
**Hold**	4°C	Hold	Hold

Sequencing and species identification

All the sequencing was performed by the LGC Genomics GmbH group. The identification was obtained by comparing the overlapping nucleotide sequences of the forward and reverse reads in

the NCBI database using the BLAST Search. The nucleotide sequences were submitted to the NIH genetic sequence database (GenBank, NCBI).

Table S4. Molecular identification of the dermatophytes isolates and corresponding accession number.

Samples ID	Organisms	Highest similiarity	% identity	Acc. Number
CV3	*T. soudanense*	e.g. MN691069.2	100	OR168629
CV4	*T. soudanense*	e.g. MN691058.2	100	OR185564
CV8	*T. soudanense*	e.g. MN691058.2	100	OR206379
CV10	*T. soudanense*	e. g. MN691070.1	100	OR185566
CV11	*T. soudanense*	e.g. MN691058.2	100	OR185565
CV12	*T. soudanense*	MN691069.2	100	OR185567
CV15	*T. soudanense*	e.g. MN691069.2	100	OR206381
CV20	*T. soudanense*	e.g. MN691069.2	100	OR185597
CV24	*T. soudanense*	MN691069.2	100	OR185598
CV30	*T. soudanense*	MN691069.2	100	OR185599
CV42	*T. soudanense*	e.g. MN691058.2	100	OR185600
CV45	*T. soudanense*	e.g. MN691057.2	99.81	OR185601
CV47	*T. soudanense*	e.g. MN691070.2	100	OR185602
CV50	*T. soudanense*	MN691069.2	100	OR185603
CV52	*T. soudanense*	MN691069.2	100	OR185607
CV55	*T. rubrum*	e.g. MT623559.1	100	OR249962
CV58	*T. soudanense*	e.g. MN691069.2	100	OR186345
CV60	*T. soudanense*	e.g. MN691058.2	100	OR206380
